# Photoelectrochemical Determination of Cardiac Troponin I as a Biomarker of Myocardial Infarction Using a Bi_2_S_3_ Film Electrodeposited on a BiVO_4_-Coated Fluorine-Doped Tin Oxide Electrode

**DOI:** 10.3390/bios13030379

**Published:** 2023-03-13

**Authors:** Thatyara Oliveira Monteiro, Antônio Gomes dos Santos Neto, Alan Silva de Menezes, Flávio Santos Damos, Rita de Cássia Silva Luz, Orlando Fatibello-Filho

**Affiliations:** 1 Department of Chemistry, Federal University of São Carlos, São Carlos 13565-905, SP, Brazil; 2 Department of Chemistry, Federal University of Maranhão, São Luís 65080-805, MA, Brazil; 3 Department of Physics, Federal University of Maranhão, São Luís 65080-805, MA, Brazil

**Keywords:** cTnI, PEC immunosensor, bismuth vanadate, bismuth sulfide

## Abstract

A sensitive and selective label-free photoelectrochemical (PEC) immunosensor was designed for the detection of cardiac troponin I (cTnI). The platform was based on a fluorine-doped tin oxide (FTO)-coated glass photoelectrode modified with bismuth vanadate (BiVO_4_) and sensitized by an electrodeposited bismuth sulfide (Bi_2_S_3_) film. The PEC response of the Bi_2_S_3_/BiVO_4_/FTO platform for the ascorbic acid (AA) donor molecule was approximately 1.6-fold higher than the response observed in the absence of Bi_2_S_3_. The cTnI antibodies (anti-cTnI) were immobilized on the Bi_2_S_3_/BiVO_4_/FTO platform surface to produce the anti-cTnI/Bi_2_S_3_/BiVO_4_/FTO immunosensor, which was incubated in cTnI solution to inhibit the AA photocurrent. The photocurrent obtained by the proposed immunosensor presented a linear relationship with the logarithm of the cTnI concentration, ranging from 1 pg mL^−1^ to 1000 ng mL^−1^. The immunosensor was successfully employed in artificial blood plasma samples for the detection of cTnI, with recovery values ranging from 98.0% to 98.5%.

## 1. Introduction

Cardiovascular diseases, especially those that present with acute morbidity, are considered one of the leading causes of mortality worldwide. Acute myocardial infarction (AMI) is the main concern, defined by pathology as myocardial necrosis due to a prolonged reduction of blood supply to the heart. It is estimated that by 2030, approximately 23.3 million people globally will die annually from AMI [1,2]. In this sense, AMI requires an early, rapid, and effective diagnosis to improve the survival rate and ensure the health quality of patients. Among the known biochemical markers of early AMI, cardiac troponin I (cTnI) is considered the golden standard in medical diagnosis [3]. In addition to being a protein specifically related to myocardial damage, it can also remain in the myocardial tissue for a long time and is released from the cells in levels of a very low concentration within 3–4 h after the onset of AMI symptoms [4,5]. For these reasons, a sensitive method for detecting cTnI is reasonable. Several reliable, sensitive, and robust methods for detecting cTnI have been proposed, including methods based on fluorescence microscopy [6], 2D-chromatography [7], colorimetry [8], surface plasmon resonance (SPR) [9,10], liquid chromatography–tandem mass spectrometry [11], and SERS-based immunoassays [12]. However, some previously proposed methods may be time-consuming, exploit labeled probes, require trained personnel, or require expensive facilities to implement, making their use in point-of-care testing difficult [6,8,9,10,11,12].

Photoelectrochemical (PEC) immunoassay has attracted growing attention in recent years [13,14,15]. This approach exploits a combination of the high sensitivity of the PEC bioanalysis and the affinity of the antigen/antibody molecules [14]. In principle, a PEC immunosensor can be easily designed using a photoelectroactive material such as a semiconductor as the signal-producing transducer and an antibody as the biological recognition element [14]. PEC measurements can be performed by employing the signal-off strategy in which the photocurrent is decreased while the formation of antibody–antigen conjugates occurs, blocking the transport of redox probes to the sensing surface (such as a label-free immunosensor) [3]. Thus, the high photoresponsive sensitivity of the semiconductor materials on the electrode is an essential aspect for the successful application of these devices.

Some photoactive semiconductor materials present a good biocompatibility, rapid reactivity, and the rapid generation and separation of electron–hole pairs [15]. Among these semiconductors, bismuth vanadate (BiVO_4_) is a promising material. Bismuth vanadate is an n-type semiconductor that presents a commonly monoclinic crystalline structure with good photocatalytic activity and a bandgap energy of 2.4 eV. It is appropriate to production of charge carriers under visible light irradiation [16]. In order to improve its photoelectrochemical efficiency and reduce the recombination processes of BiVO_4_, heterojunctions commonly based on semiconductors with a narrower bandgap could be employed, such as bismuth sulfide (Bi_2_S_3_). Bismuth sulfide also is an n-type semiconductor and has a bandgap of 1.3 eV. Bi_2_S_3_ presents a reasonable efficiency of photocurrent conversion under visible light [17] and has become attractive for many PEC applications [18,19,20,21]. In this paper, based on the properties of the BiVO_4_ and Bi_2_S_3_ materials described herein, we report a label-free PEC immunosensor designed with a junction of these two semiconductors to determine the cTnI biomarker in clinical samples in real time, exploiting the effects of the immunoreaction upon the response of the PEC platform to the ascorbic acid (AA) donor molecule.

## 2. Materials and Methods

### 2.1. Reagents and Chemicals

Human cardiac troponin I (cTnI), monoclonal cTnI antibody (anti-cTnI), N’-ethylcarbodiimide hydrochloride (EDC), N-hydroxysuccinimide (NHS), bovine serum albumin (BSA), bismuth nitrate (Bi(NO_3_)_3_), ammonium metavanadate (NH_4_VO_3_), ethylene glycol, and thioglycolic acid were purchased from Sigma-Aldrich (St. Louis, MO, USA). Sodium thiosulfate, ethylenediaminetetraacetic acid disodium salt dihydrate (C_10_H_14_N_2_Na_2_O_8_ · 2H_2_O), sodium hydroxide, sodium dihydrogen phosphate, disodium hydrogen phosphate, ascorbic acid, citric acid, acetic acid, boric acid, phosphoric acid, sodium chloride, potassium chloride, calcium chloride, ammonium chloride, disodium sulfate, potassium dihydrogen phosphate, and urea were acquired from ISOFAR (Duque de Caxias, RJ, Brazil). All aqueous solutions were prepared with water purified in a OS10LXE Gehaka osmose system (São Paulo, SP, Brazil).

### 2.2. Experimental Apparatus

Photoelectrochemical experiments were performed using an Autolab potentiostat/galvanostat model PGSTAT 128N (Metrohm Autolab B. V., Netherlands) equipped with a Frequency Response Analyzer module, controlled by NOVA software, and coupled to a three-electrode electrochemical cell confined in a box to control the illumination on the photoelectrodes. A commercial 36 W LED lamp was used as a visible light source. A FTO glass photoelectrode (5 cm length × 1 cm width, with a modified area of 0.7 × 1.0 cm^2^), modified with Bi_2_S_3_/BiVO_4_, was used as the working electrode. Ag/AgCl/KCl_sat_) was used as the reference electrode, and a Pt wire was used as the counter electrode. Electrochemical impedance spectroscopy experiments were carried out in a 0.1 mol L^−1^ KCl solution containing 5 mmol L^−1^ K_3_[Fe(CN)_6_] in the frequency range of 10^−1^–10^5^ Hz under an AC amplitude of 10 mV.

### 2.3. Synthesis of BiVO_4_

This procedure was performed according to method proposed by [22]. For this step, 0.4850 g of Bi(NO_3_)_3_.5H_2_O was transferred into a falcon tube containing 5 mL of ethylene glycol and sonicated for 40 min. A mass of 0.1175 g of NH_4_VO_3_ was transferred into a falcon tube containing 5 mL of water and heated in a water bath until complete dissolution. The latter solution was then dropped slowly into the former solution under constant stirring, causing the formation of a yellow BiVO_4_ suspension.

### 2.4. Construction of Bi_2_S_3_/BiVO_4_/FTO Photoelectrochemical Sensor

Prior to modification, the FTO electrode was washed with water and ethanol to remove any adsorbed species on the surface. The FTO plates were then sustained until the temperature equilibrated at 100 °C to the deposition of the BiVO_4_ material. A volume of 35 µL of BiVO_4_ suspension was dropped into 0.7 × 1.0 cm² of the FTO heated plate and a BiVO_4_ film was formed after few minutes. This procedure was repeated three times to completely cover the FTO electrode surface. The BiVO_4_/FTO platform was annealed at 500 °C for 1 h using an oven with a heating rate of 10 °C min^−1^. After annealing, the BiVO_4_/FTO electrode was allowed to cool to room temperature. Next, a Bi_2_S_3_ film was electrodeposited onto the BiVO_4_/FTO platform, according to the method proposed by [23] with few adaptations. An amount of 0.04 mol L^−1^ Na_2_S_2_O_3_ was diluted in a solution containing 4.5 mL purified water and 0.5 mL HCl. After the formation of colloidal sulfur, the solution was kept still at room temperature for 24 h to allow the sulfur particles to precipitate at the bottom of the solution. The supernatant solution was then used for the preparation of another solution containing 0.006 mol L^−1^ Bi(NO_3_)_3_ and 0.006 mol L^−1^ EDTA. The Bi_2_S_3_ electrodeposition onto the BiVO_4_/FTO electrode surface was then performed by amperometry, applying a potential of −0.9 V vs. Ag/AgCl/KCl_sat_ for 300 s at room temperature. The Bi_2_S_3_/BiVO_4_/FTO platform was annealed using a hot plate at 200 °C for 30 min.

### 2.5. Optimization of the Platform Response for the AA before and after the Immobilization of the Biological Materials

Some experimental parameters were optimized to achieve a higher sensitivity of the Bi_2_S_3_/BiVO_4_/FTO PEC platform to the AA donor molecule. Thus, the effect of the applied potential on the sensor response was initially investigated by evaluating the photocurrents in 0.03 mol L^−^^1^ AA in a 0.1 mol L^−1^ phosphate buffer, at pH 7.4 under different potentials (from −0.2 V to 0.2 V vs. Ag/AgCl/KCl_sat_). The influence of the nature of the buffer solution on the response of the platform was then evaluated by monitoring the photocurrents obtained from 0.03 mol L^−^^1^ AA under an applied potential of 0 V vs. Ag/AgCl/KCl_sat_ in three different buffer solutions: phosphate, McIlvaine, and Britton–Robinson. The study of the effects of the buffer solutions was performed by maintaining the buffer concentration at 0.1 mol L^−^^1^ and pH 7.4. The effect of the concentration of the AA donor molecule on the response of the platform was also investigated for AA concentrations from 0.01 to 0.06 mol L^−^^1^.

After the optimization of the Bi_2_S_3_/BiVO_4_/FTO PEC platform response for the concentration of the AA molecule, the platforms were modified with the antibody of troponin (anti-cTnI). Initially, the electrode was incubated in an aqueous solution of 0.003 mol L^−^^1^ thioglycolic acid (TGA) at room temperature for 15 min to introduce carboxylic groups at the surface of the Bi_2_S_3_/BiVO_4_/FTO PEC platform. Following this, the excess TGA was removed with water and 15 µL of an EDC/NHS mixed solution (0.15 mol L^−^^1^ EDC and 0.10 mol L^−^^1^ NHS) was added and incubated for 1 h on the functionalized surface to activate the -COOH groups. The activated platform was then incubated with 10 µL of different concentrations of anti-cTnI (from 1 to 5 µg mL^−^^1^) at room temperature for a determinate time. The platform modified with the antibody was gently washed to remove the weakly adsorbed anti-cTnI. Furthermore, the EDC/NHS excess was removed from surface with purified water. Next, 10 µL of 1 % (*m*/*v*) BSA solution was added to block nonspecific binding sites. Posteriorly, the effect of the interaction time between the antibody and the antigen on the response of AA (10, 15, 20, 25, and 30 min) was studied using a concentration of 1 ng mL^−^^1^ cTnI. After evaluating all the experimental parameters, the conditions that provided the highest photocurrent value were fixed to finally obtain the analytical curve for the determination of cTnI. The analytical curve was obtained after the incubation of the anti-cTnI/Bi_2_S_3_/BiVO_4_/FTO PEC immunosensor platform with different cTnI concentrations (from 1 pg mL^−^^1^ to 1000 ng mL^−^^1^ cTnI).

### 2.6. Preparation and Analysis of Artificial Blood Plasma Samples

The performance of the anti-cTnI/Bi_2_S_3_/BiVO_4_/FTO PEC immunosensor was evaluated by the determination of cTnI in artificial blood plasma samples using an external calibration method. The artificial samples were composed of 0.138 mol L^−1^ NaCl, 0.004 mol L^−1^ NaHCO_3_, 0.003 mol L^−1^ KCl, 0.001 mol L^−1^ Na_2_HPO_4_ · 3H_2_O, 0.002 mol L^−1^ MgCl_2_ · 6H_2_O, 0.003 mol L^−1^ CaCl_2_, and 0.507 mmol L^−1^ Na_2_SO_4_ [24]. The samples were spiked with different concentrations of cTnI (0.05, 2.0 and 50 ng mL^−1^), and aliquots of 10 µL of each sample were added directly onto the immunosensor surface at the optimal conditions and analyzed in presence of the AA molecule using the developed sensor in three replicates.

### 2.7. X-ray Diffraction and Scanning Electron Microscopy

X-ray Diffraction (XRD) measurements were obtained with a D8 Advance diffractometer (Bruker) equipped with the LynxEye linear detector, using Cu Ka radiation (k = 1.5418 Å) and operating at 40 kV/40 mA. The diffraction patterns were collected from 15 to 80 ^o^ with a step size of 0.02^o^ and a counting time of 0.6 s. The sample surface was imaged on an EVO HD electron microscope (Zeiss) at 10 kV after being fixed onto stubs using a carbon film.

## 3. Results and Discussion

### 3.1. Characterization of the Materials by X-Ray Diffraction and Scanning Electron Microscopy

**Figure 1:**
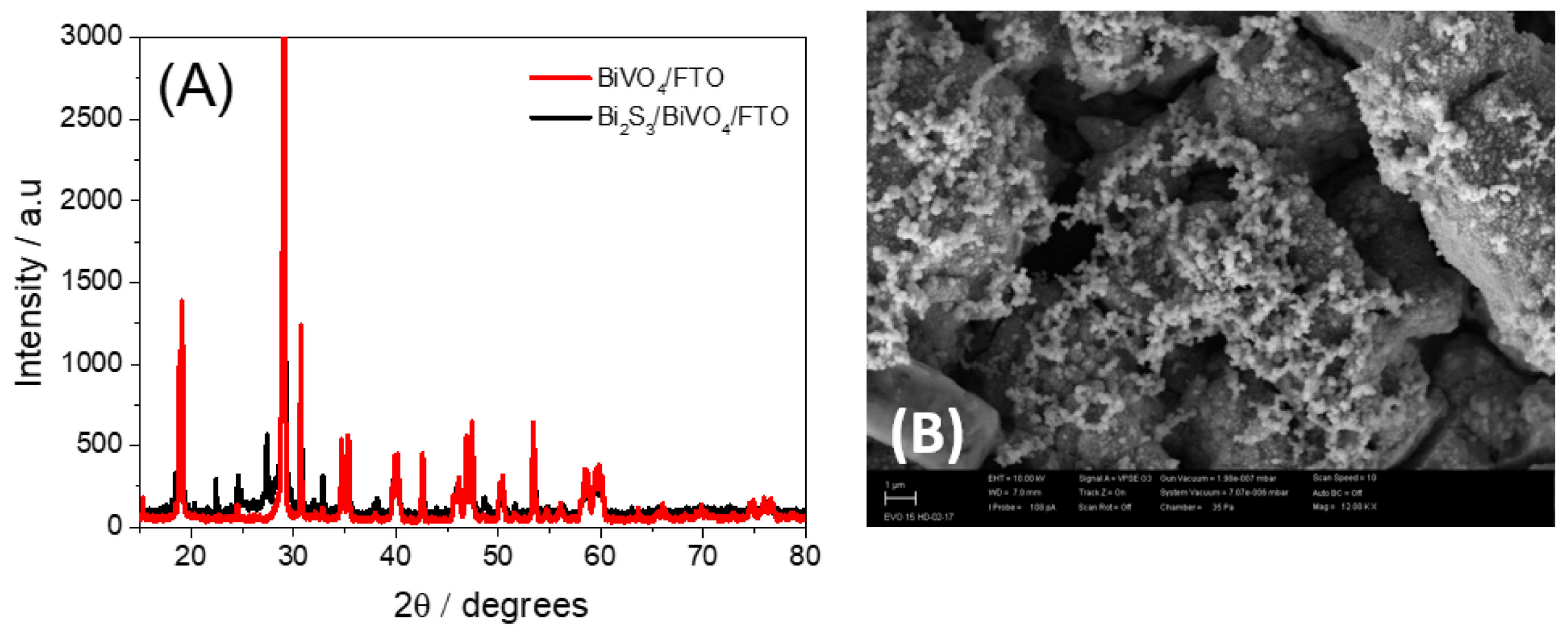
(**A**) X-Ray diffraction patterns of BiVO_4_/FTO and BiVO_4_/Bi_2_S_3_/FTO. (**B**) SEM image of BiVO_4_/Bi_2_S_3_/FTO. Scale bar = 1 µm.

The materials utilized for the construction of the photoelectrochemical platform were evaluated by X-ray diffraction (XRD). In Figure 1A (red line), the characteristic peaks of the BiVO_4_/FTO can be observed. The 2θ values of BiVO_4_ at 18.7, 28.7, 30.4, 34.5, 35.2, 39.7, 42.2, 46.7, 50.3, 53.3, and 58.4 correspond to the (110)(011), (−130)(−121)(121), (040), (200), (002), (211), (051), (240), (202), (−161)(161), and (−321)(321) planes of the monoclinic BiVO_4_ scheelite (JCPDS 014-0688) [16]. A small amount of the BiVO_4_ tetragonal phase (JCPDS 014-0133) was also detected, and its most intense peak (200) can be seen at 24.4°. Furthermore, additional diffraction peaks appear after the Bi_2_S_3_ electrodeposition, as can be seen in Figure 1A (black diffractogram). In this figure, the XRD spectrum of the BiVO_4_/Bi_2_S_3_/FTO photoelectrochemical platform shows the characteristic diffraction peaks of the BiVO_4_/FTO platform. In addition, one can see a peak at 27.2° from the (012) plane of the Bi rhombohedral phase and the (220) and (130) peaks (at 22.3 and 24.8, respectively) of the orthorhombic Bi_2_S_3_ phase (JCPDS 017-0320), clearly demonstrating that the modification process was effective and that all samples display a high crystallinity. In addition, to show the morphology of the Bi_2_S_3_/BiVO_4_/FTO platform, a scanning electron microscopy (SEM) image was obtained. Figure 1B shows smaller particles covering an agglomerated material. These smaller particles resulted from the electrodeposition of Bi_2_S_3_ on the BiVO_4_/FTO platform surface after the preparation procedure of the Bi_2_S_3_/BiVO_4_/FTO electrochemical platform, which was described in the experimental section.

### 3.2. EIS and Photoelectrochemical Characterization of Bi_2_S_3_/BiVO_4_/FTO Platform

Electrochemical impedance spectroscopy (EIS) is a powerful tool for monitoring the charge transfer resistance, which can be used to demonstrate the successful fabrication of a PEC-sensing platform [25]. EIS measurements were taken to evaluate the charge transfer characteristics of the semiconductor materials. EIS measurements were also performed to evaluate the electrocatalytic effect of the semiconductor materials and the increase in the electroactive area of the working electrodes.

**Figure 2:**
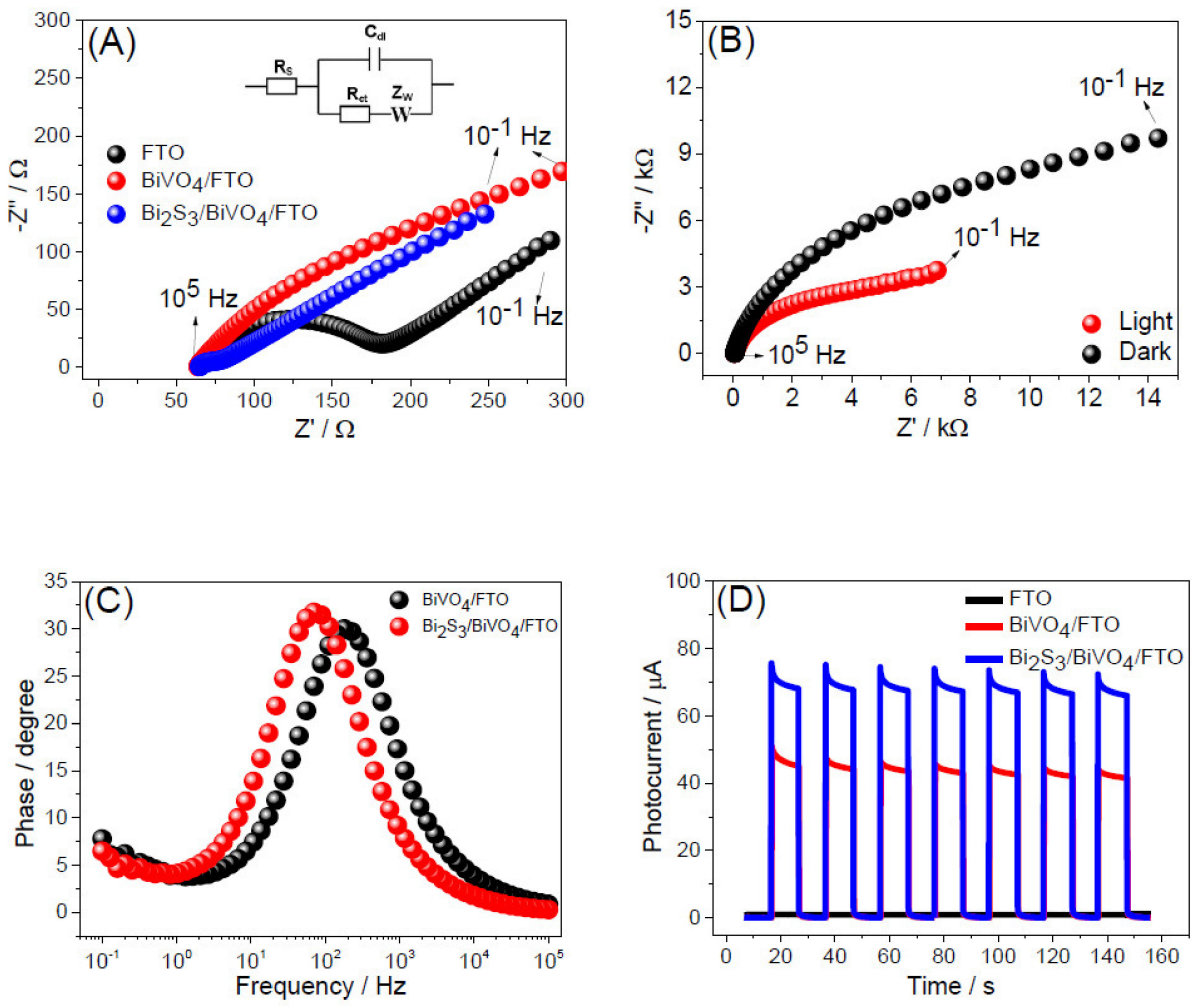
(**A**) Nyquist plots for the FTO (black), BiVO_4_/FTO (red), and Bi_2_S_3_/BiVO_4_/FTO (blue) electrodes, recorded in 0.1 mol L^−1^ KCl solution containing 5 mmol L^−1^ Fe[(CN_6_)]^3-/4-^. (**B**) Nyquist plots for the Bi_2_S_3_/BiVO_4_/FTO photosensor in aqueous 0.1 mol L^−1^ Na_2_SO_4_ solution, recorded at an open-circuit potential in the dark (black) and under the visible LED light irradiation (red). (**C**) Bode phase plots for BiVO_4_/FTO (black) and Bi_2_S_3_/BiVO_4_/FTO (red) electrodes in 0.1 mol L^−1^ KCl solution containing 0.03 mol L^−1^ AA donor molecules. (**D**) Photocurrent response of FTO (black line), BiVO_4_/FTO (red line), and Bi_2_S_3_/BiVO_4_/FTO (blue line) photoelectrodes. Amperometric measurements were performed in a 0.1 mol L^−1^ phosphate buffer (pH 7.4) containing 0.03 mol L^−1^ AA, E_appl_ = 0 V vs. Ag/AgCl/KCl_sat_.

Figure 2A shows the Nyquist plots of the FTO, BiVO_4_/FTO, and Bi_2_S_3_/BiVO_4_/FTO electrodes, recorded at the open circuit potential and modeled using an adapted Randles equivalent circuit (inset in Figure 2A) consisting of a cell resistance (R_s_) in series with a parallel combination of a constant phase element (CPE), considered a non-ideal capacitance, and a charge transfer resistance (R_ct_) with a Warburg impedance (Z_w_). Following the fitting of the parameters presented in Figure 2A. The BiVO_4_/FTO electrode presented a charge transfer resistance value of 515 Ω, while the Bi_2_S_3_/BiVO_4_/FTO electrode exhibited an R_ct_ value of only 93 Ω. These results suggest that the sensitization of the platform by Bi_2_S_3_ enhanced the electron transfer in the electrode–solution interface, contributing to the improved electrocatalytic properties of the sensing platform. Furthermore, the capacitance values of the BiVO_4_/FTO and Bi_2_S_3_/BiVO_4_/FTO electrodes were determined to be 0.0629 mF and 3.08 mF, respectively. These results indicate an increase in the electroactive area of the surface due to increase in the capacitances.

The effects of the LED light on the electron transfer processes and the lifetime of the electron were evaluated for the different sensing platforms to investigate the nature of their PEC responses. Figure 2B presents the Nyquist plots of the Bi_2_S_3_/BiVO_4_/FTO electrode obtained in 0.1 mol L^−1^ Na_2_SO_4_ solution in the absence and in the presence of visible LED light. As can be seen, there was a decrease in the semicircle diameter of spectra in the presence of LED light, indicating that the R_ct_ value decreased. These results suggest that light irradiation enhances the electrocatalytic potential of the sensing platform since it enables the formation of electron–hole pairs in the Bi_2_S_3_/BiVO_4_ composite material. Bode phase plots were obtained for the BiVO_4_/FTO and Bi_2_S_3_/BiVO_4_/FTO electrodes in order to estimate the lifetime of the electron through the following equation [26]:

(1) τe=12πfmax

where $\tau_{e}$ is the lifetime of the electron and *f_max_* is the maximum frequency in the Bode phase diagram.

Figure 2C shows that the *f_max_* in the Bode phase plots of the BiVO_4_ platform decreased from 179.6 Hz to 70.38 Hz after sensitization by Bi_2_S_3_, reflecting the increase in the lifetime of the electron from 0.886 ms to 2.26 ms. The enhancement of the lifetime of the electron suggests that the formation of the Bi_2_S_3_/BiVO_4_ heterojunction enabled a slower recombination of the electron–hole pairs, which also explains the more favorable electron transfer between composite material and FTO electrode, as shown in the study of Figure 2A.

Additionally, the photocurrent intensity obtained for each individual component of the developed PEC platform was evaluated in the presence of an AA donor molecule (Figure 2D). As can be seen, there was an increase in the photocurrent response for the BiVO_4_/FTO platform after sensitization by Bi_2_S_3_ film, confirming an improvement in the photo–current conversion efficiency due to a greater absorption of visible light, an increase in the electroactive area, and an improvement in the electrochemical properties of the platform. Although BiVO_4_ is a semiconductor with good photocatalytic activity, its photoelectrochemical efficiency may not reach a desirable level due to the occurrence of charge recombination [27], which is minimized after the conjunction of energy bands in the Bi_2_S_3_/BiVO_4_ composite. Furthermore, Bi_2_S_3_ is a material that presents a high surface activity, further improving the catalytic activity of the PEC platform [28].

### 3.3. Evaluation of Experimental Parameters on the Bi_2_S_3_/BiVO_4_/FTO PEC Platform Response

Initially, the effect of the applied potential, buffer type, and AA (electron donor molecule) concentration on the response of the Bi_2_S_3_/BiVO_4_/FTO PEC platform were evaluated. The applied potential is an important parameter that can directly influence the analytical performance of a sensor. Thus, the effect of the applied potential on the response of the Bi_2_S_2_/BiVO_4_/FTO PEC platform was investigated, and the results are presented in Figure S1. According to this figure, it can be observed that the photocurrent of the Bi_2_S_2_/BiVO_4_/FTO PEC platform in the presence of 0.03 mol L^−1^ AA increased when the applied potential changed from −0.2 V to 0 V vs. Ag/AgCl/KCl_sat_, and it remained almost constant from 0 to 0.1 V vs. Ag/AgCl/KCl_sat_. These results suggest that using a potential of 0 V is enough to obtain a high photocurrent value. Under these conditions, a higher sensitivity to the system was achieved while maintaining low biasing conditions, making it possible to determine the analyte even at very low concentrations that consume a minimum of energy. In addition, it is possible to significantly reduce or eliminate the possible influence of interfering species on the photoelectrochemical processes. At potentials above 0.1 V, it can be observed that the photocurrent tended to decrease. This can be related to a lower stability of the Bi_2_S_2_/BiVO_4_ film, causing a lower efficiency for AA oxidation. In this sense, an applied potential of 0 V was chosen to construct the analytical curve for the determination of the antioxidant; all subsequent measurements were then performed under 0 V.

Posteriorly, the effect of the following buffer solutions: phosphate, McIlvaine, and Britton–Robinson on the response of the Bi_2_S_2_/BiVO_4_/FTO PEC platform in the presence of 0.03 mol L^−1^ AA were evaluated (Figure S2). Figure S2 showed no significative difference in the intensity of photocurrent among the different electrolytes studied; however, considering the high stability of the AA photocurrent response and the simplicity of preparing the buffer, a phosphate buffer solution was chosen for all subsequent assays of the PEC sensor. Furthermore, the effect of the donor molecule concentration on the platform response was also investigated by monitoring the photocurrent of the platform for AA in the following concentrations: 0.01, 0.02, 0,03, 0.04, 0.05, and 0.06 mol L^−1^ (Figure S3). This study showed that the process of donor molecule oxidation achieves maximum efficiency when the AA concentration reaches 0.04–0.05 mol L^−1^. Above this concentration range, there is a tendency to obtain a lower photocurrent because the PEC platform hinders the oxidation of the analyte. In this context, the donor molecule concentration was kept at 0.04 mol L^−1^ for all subsequent studies. Subsequently, the influence of the concentration of the immobilized anti-cTnI antibodies on the Bi_2_S_3_/BiVO_4_/FTO PEC platform was evaluated, as was the interaction time between the antibody and the antigen (cTnI) immobilized on the PEC platform. Both studies were performed in the presence of 0.04 mol L^−1^ AA.

**Figure 3:**
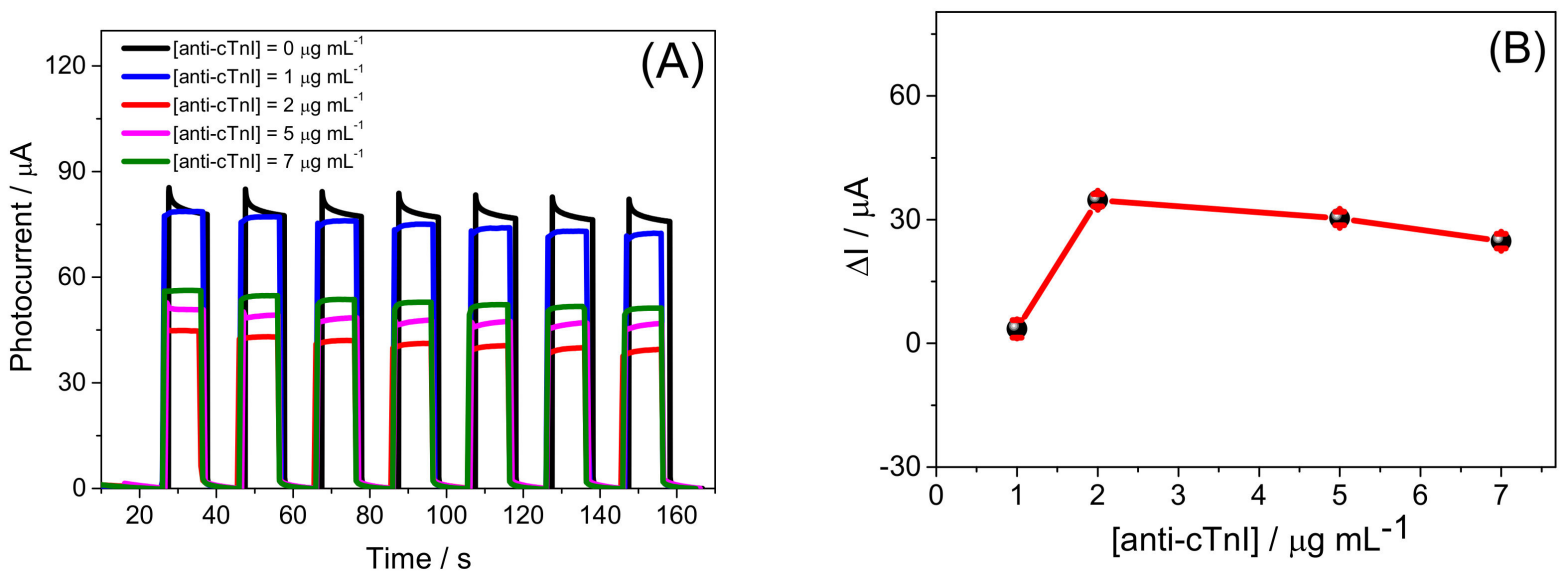

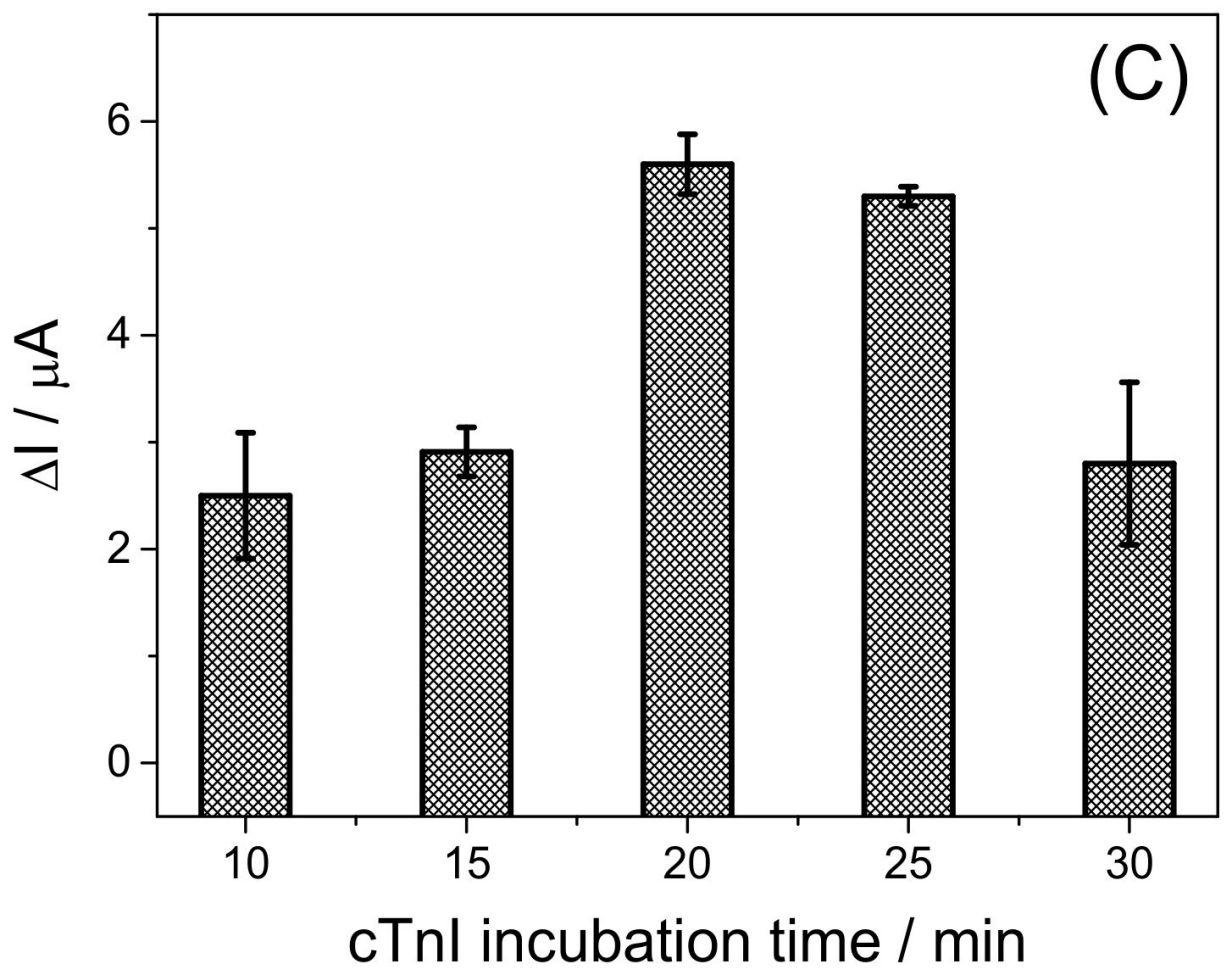
(**A**) Photoelectrochemical response of the Bi_2_S_3_/BiVO_4_/FTO PEC platform for different anti-cTnI concentrations and (**B**) plot of the photocurrent variation vs. [anti-cTnI]. Data obtained from Figure 3(**A**). (**C**) Effect of incubation time of the anti-cTnI/Bi_2_S_3_/BiVO_4_/FTO PEC platform with 1 ng mL^−1^ cTnI on the variation of the photocurrent. All measurements were carried in 0.1 mol L^−1^ phosphate buffer, pH 7.4, containing 0.04 mol L^−1^ AA. E_appl_ = 0 V vs. Ag/AgCl/KCl_sat_.

Figure 3A shows the photoelectrochemical response of the Bi_2_S_3_/BiVO_4_/FTO PEC platform in a phosphate buffer containing 0.04 mol L^−1^ AA after the incubation of the platform with different concentrations of anti-troponin I (anti-cTnI) (1, 2, 5 and 7 µg mL^−1^). Figure 3B shows the variation of the photocurrents (ΔI = I_0_ − I, where I_0_ and I are the photocurrents obtained before and after incubation of the platform with anti-cTnI, respectively) obtained from Figure 3A. According to Figure 3B, it can be observed that the photocurrent presented a high increase from 1 to 2 µg mL^−1^. At concentrations of 2, 5, and 7 µg mL^−1^, the photocurrent presented a percentage of decrease in relation to the initial photocurrent value, I_0_ (without antibody immobilization) of approximately 50, 40, and 35%, respectively. Based on these results, it was considered that any of the three concentrations (2, 5, or 7 µg mL^−1^) could be used for the preparation of the anti-cTnI/Bi_2_S_3_/BiVO_4_/FTO photoelectrochemical immunosensor. In this context, an intermediate concentration of 5 µg mL^−1^ was chosen for further experiments.

In order to evaluate the effects of the interaction time between the antibodies (anti-cTnI) immobilized on the platform and the antigens (cTnI) from the incubation solution, the immunosensor platform was incubated with 1 ng mL^−1^ of cTnI antigens in a phosphate buffer solution containing 0.04 mol L^−1^ AA at several incubation times (10, 15, 20, 25, and 30 min). Figure 3C shows the variation of the photocurrent of the anti-cTnI/Bi_2_S_3_/BiVO_4_/FTO PEC immunosensor before, I_0_, and after, I, the interaction with the cTnI antigens (ΔI = I_0_ − I). According to the results shown in Figure 3C, the inhibition of the photocurrent of the anti-cTnI/Bi_2_S_3_/BiVO_4_/FTO PEC immunosensor to the donor molecule increased significantly when the incubation time increased from 10 min to 20 min, with a low decrease observed for 25 min of interaction. As the standard deviation of the photocurrent was lower at 25 min, this interaction time between the antibodies and the Troponin I antigens was selected for all subsequent assays. The amperograms shown in Figure 3C are presented in Figure S4 in the supporting information. Additionally, it is important to emphasize that the results presented in this figure suggest that it is possible to monitor the cTnI antigen concentrations with the anti-cTnI/Bi_2_S_3_/BiVO_4_/FTO PEC immunosensor from the decrease in the analytical signal. However, in order to propose a possible mechanism for the detection of troponin I, the effects of different concentrations of cTnI on the variation of photocurrent of the immunosensor were evaluated.

### 3.4. Analytical Performance of Bi_2_S_3_/BiVO_4_/FTO PEC Immunosensor

**Figure 4:**
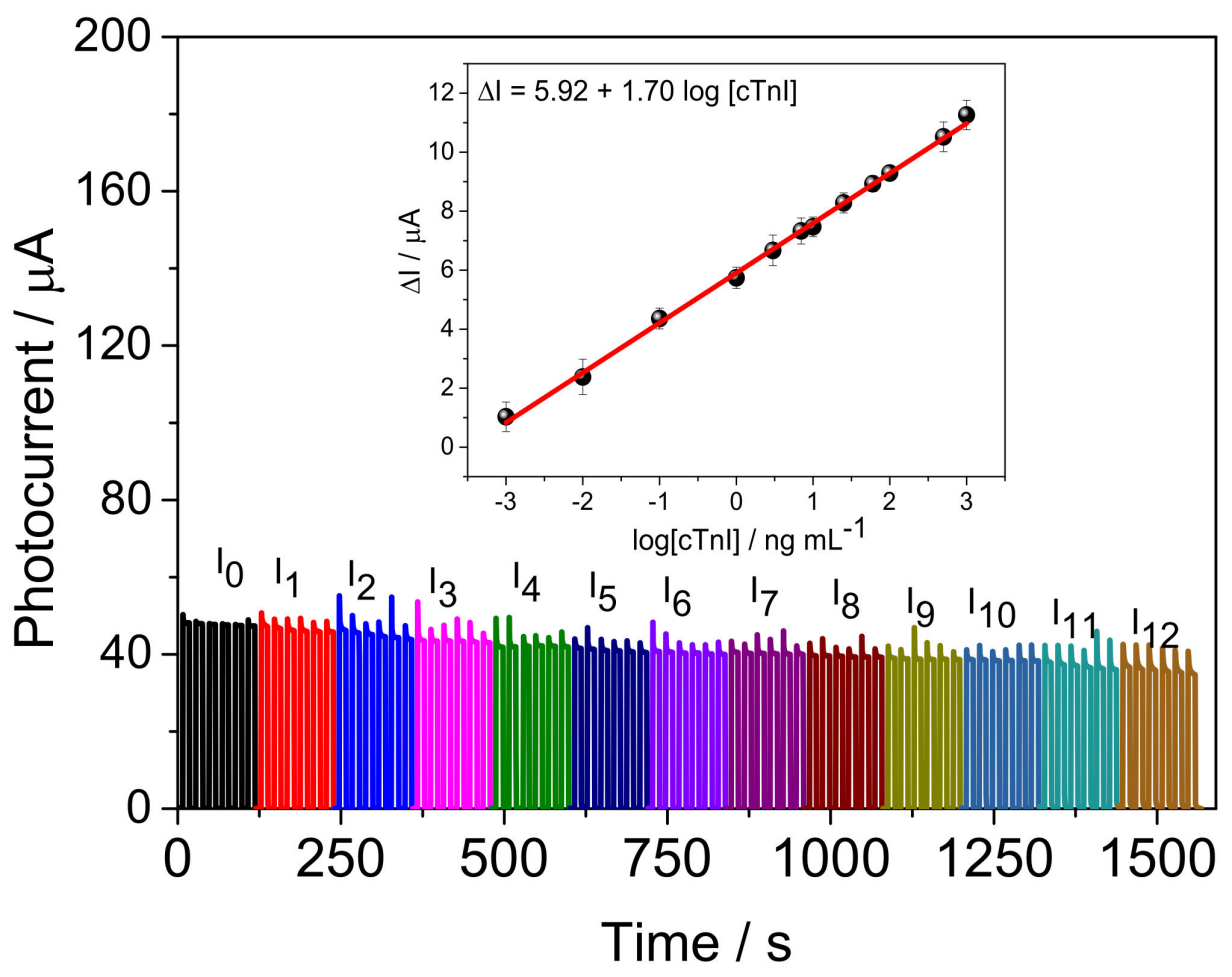
Photoelectrochemical response of the anti-cTnI/Bi_2_S_3_/BiVO_4_/FTO immunosensor after incubation with different cTnI concentrations. [AA]= 0.04 mol L^−1^. Inset: Analytical curve obtained from of the data of Figure 4. [cTnI] from of 1 pg mL^−1^ to 1000 ng mL^−1^. E_appl_ = 0 V vs. Ag/AgCl/KCl_sat_.

Under the optimum immunoassay conditions, the PEC platform response to the AA donor molecule was evaluated after incubation with different cTnI concentrations. Figure 4 exhibits the photocurrent responses for different concentrations. The immunocomplex formation on the platform surface decreased the AA photocurrent, whose inhibition is expressed in ΔI values (ΔI = I_0_ − I_n_) in which I_0_ and I_n_ correspond to the AA photocurrent before and after interaction with the immunosensor with cTnI, respectively. As can be seen in the inset of Figure 4, an analytical curve was obtained for the concentration range from 1 pg mL^−1^ to 1000 ng mL^−1^ cTnI, linear equation for which was ΔI (µA) = 5.92 (±0.04) + 1.70 (±0.02) log[cTnI] (ng mL^−1^), with the correlation coefficient of 0.998 (*n* = 12). An experimental limit of detection (LOD) of 1 pg mL^−1^ was obtained from a signal-to-noise ratio equal to three. The LOD obtained was significantly lower than the maximum limits allowed for a clinical diagnosis of myocardial infarction at approximately 0.1 ng mL^−1^ [29]. In combination with the linear range of response, this result was compared to further reported PEC immunosensors for cTnI (Table 1: references [3,15,24,25,26,27,28,29,30,31,32,33,34,35,36,37,38,39]). As can be seen, the anti-cTnI/Bi_2_S_3_/BiVO_4_/FTO immunosensor presents some interesting features for the determination of this biomarker in comparison to previously reported PEC sensors.

**Scheme 1:**
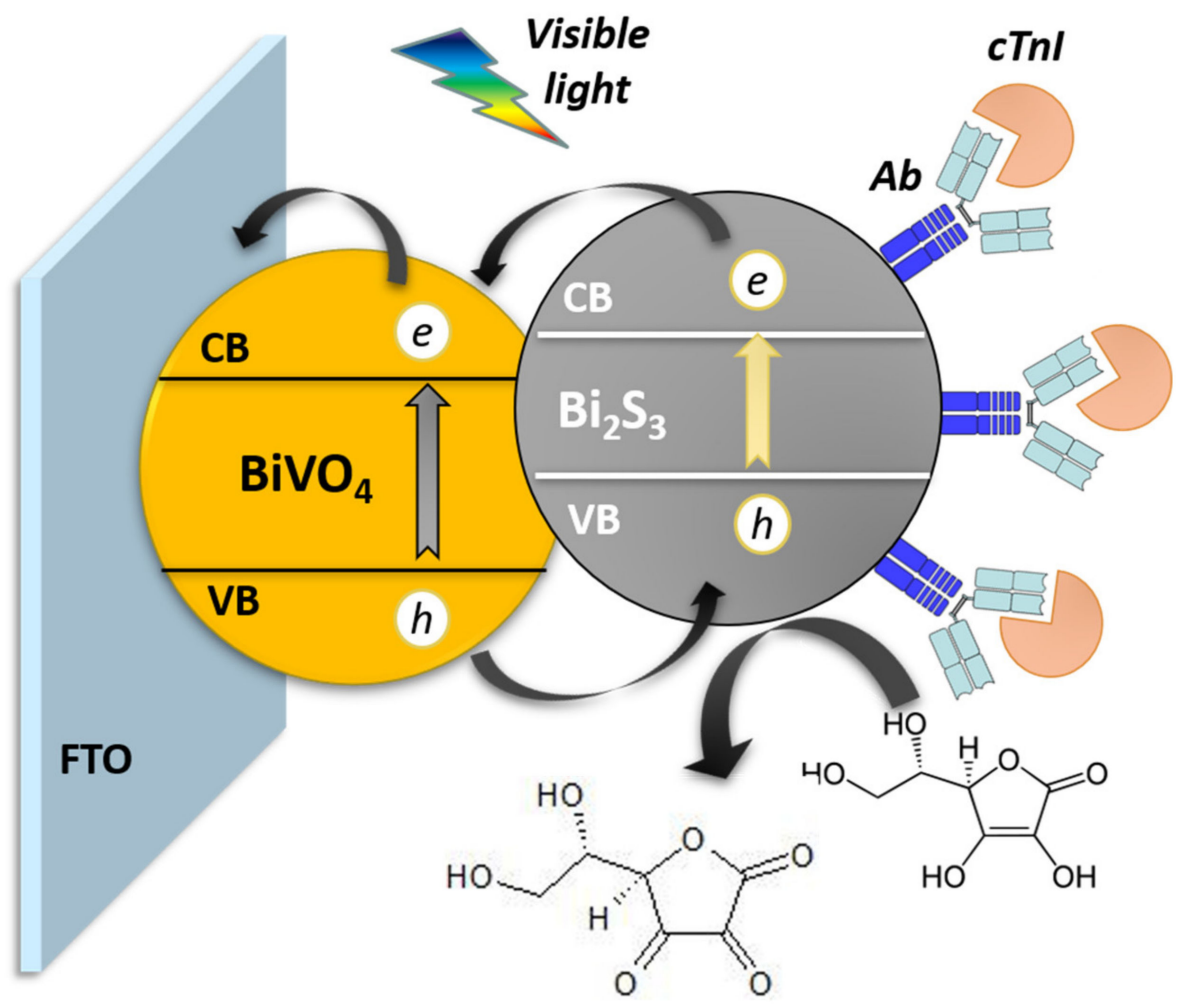
Representation of the proposed mechanism for PEC determination of cTnI by label-free Bi_2_S_3_/BiVO_4_/FTO immunosensor. Ab: anti-cTnI.

Considering the anti-cTnI/Bi_2_S_3_/BiVO_4_/FTO PEC immunosensor response to the decrease in the AA molecule with the increase in the cTnI antigen concentration, a schematic representation of the PEC determination of cTnI (Scheme 1) under the incidence of light was proposed. As shown in Scheme 1, the BiVO_4_ and Bi_2_S_3_ harvest photons of energy higher than their band gap, promoting electrons from the valence to the conduction band and giving rising to e^−^/h^+^ couples. The electron photogenerated at the conduction band of Bi_2_S_3_ can be injected into the conduction band of the BiVO_4_, while the hole photogenerated in the valence band of the Bi_2_S_3_ can be transferred to the AA molecule. The AA molecule acts as an ideal electron donor to capture the photogenerated holes in the valence band (VB) of Bi_2_S_3_, inhibiting the recombination of electron–hole pairs [30] and generating an anodic photocurrent. The cTnI biomarker can then interact with the immobilized anti-cTnI/Bi_2_S_3_/BiVO_4_/FTO, decreasing the efficiency of the system to produce a photocurrent since the cTnI biomarker/anti-cTnI interaction reduces the efficiency of the photoactive material to transfer holes to donor molecules, an inhibition that is proportional to the amount of cTnI antigens immobilized on the immunosensor surface.

**Figure 5:**
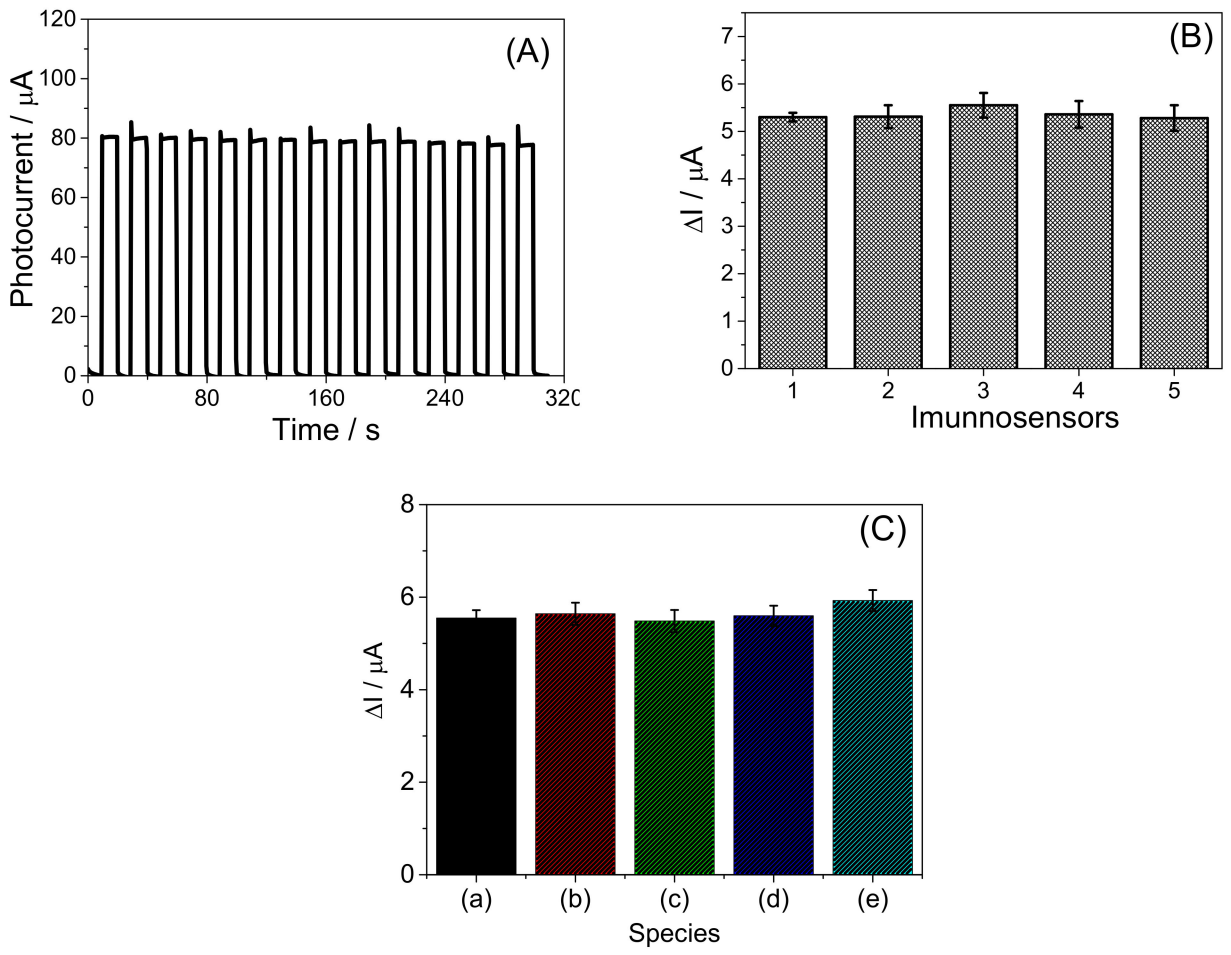
(**A**) Evaluation of repeatability of photocurrent of the Bi_2_S_3_/BiVO_4_/FTO PEC platform. (**B**) Reproducibility of photocurrent of the Bi_2_S_3_/BiVO_4_/FTO PEC platform incubated with 1 ng mL^−1^ cTnI antigen solution. (**C**) Evaluation of selectivity of the anti-cTnI/Bi_2_S_3_/BiVO_4_/FTO PEC immunosensor. (**a**) cTnI, (**b**) cTnI + albumin, (**c**) cTnI + C-reactive protein, (**d**) cTnI + glucose, and (**e**) cTnI + myoglobin. [cTnI] = 1 ng mL^−1^. [Foreign species] = 100 ng mL^−1^. Measurements performed in 0.1 mol L^−1^ phosphate buffer, pH 7.4. [AA]= 0.04 mol L^−1^, E_appl_ = 0 V vs. Ag/AgCl/KCl_sat_.

For practical application, repeatability, reproducibility, and selectivity are important features of the immunosensor [5,15]. Figure 5A presents the photocurrent response of the Bi_2_S_3_/BiVO_4_/FTO platform to AA. As can be seen, no significative photocurrent changes were observed. The relative standard deviation (RSD) among signals was only 1.04% (*n* = 15), indicating that the proposed sensing platform had good stability for the interaction with the AA donor molecule.

The reproducibility of the proposed immunosensor also was evaluated (Figure 5B). This parameter was assessed using five different electrodes for the same concentration of cTnI and under optimal experimental conditions. The RSD value obtained for this study was 2.1%, indicating a satisfactory reproducibility of the immunosensor.

In order to appraise the selectivity of the PEC immunosensor for cTnI detection, some potential interfering substances were investigated. Therefore, a solution containing 1 ng mL^−1^ cTnI (a) and solutions containing 1 ng mL^−1^ cTnI and 100 ng mL^−1^ of potential interfering substances such as albumin (b), C-reactive protein (c), glucose (d), and myoglobin (e) were tested, respectively, under optimized experimental conditions. The results are shown in Figure 5C. As can be seen, there was no significant change in the photocurrent presented by the immunosensor after the addition of those substances, and the RSD of the measurement was within 5%, indicating that the designed immunosensor possesses a remarkable selectivity for detection of cTnI. The data from Figure 5 are presented in Figures S5 and S6 in the supporting information.

### 3.5. Detection of cTnI in the Artificial Blood Plasma Samples

To demonstrate the accuracy and the potential of application of the developed immunosensor in clinical samples, the anti-cTnI/Bi_2_S_3_/BiVO_4_/FTO electrode was tested to determine cTnI at different concentrations in artificial blood plasma samples. The samples were spiked with cTnI at three concentration levels (0.05, 2.0, and 50 ng mL^−1^), and quantification performed using an external calibration method. The recovery values (Table 2) were found between 98.0% and 98.5% with very low values of relative standard deviation, indicating good accuracy and precision. Thus, the results suggest that the immunosensing platform based on Bi_2_S_3_/BiVO_4_/FTO can be used as a promising strategy for the detection of cTnI in clinical blood plasma samples.

## 4. Conclusions

In this paper, the feasibility of the sensitization of a BiVO_4_ semiconductor material with a Bi_2_S_3_ electrodeposited film for the development of a PEC immunosensing platform to determine cTnI as a biomarker of myocardial infarction was reported. The proposed immunosensor presented high photoelectrochemical efficiency under visible LED light irradiation, a wide linear range of response to cTnI, high sensitivity, a low limit of detection, and good selectivity and stability. Moreover, the immunosensor demonstrated good accuracy and good precision with excellent application for the termination of the cTnI biomarker in artificial blood plasma samples.

## Figures and Tables

**Table 1:** Comparison of the analytical parameters of different PEC sensors for detection of cTnI.

Modified Electrode	Technique	Linear Range (ng mL^−1^)	LOD (pg mL^−1^)	Reference
Bi_2_S_3_/BiVO_4_/FTO	PEC	0.0010–1000	1.0	This work
^a^ NAC-CdAgTe QDs/AuNPs/GCE	PEC	0.0050–20	1.70	[3]
^b^ Mn:CdS@Cu_2_MoS_4_/G/ITO	PEC	0.0050–1000	0.18	[15]
^c^ CM-dextran/Au/TiO_2_ NTA/Ti	PEC	0.00484–0.484	2.20	[31]
^d^ SnO_2_/NCQDs/BiOI/ITO	PEC	0.00100–100	0.30	[32]
^e^ Au NPs/ZIS/Bi_2_Se_3_/ITO-PET	PEC	0.080–40	26.00	[33]
^f^ Zn_2_SnO_4_/N,S-GQDs/CdS/ITO	PEC	0.0010–50	0.30	[34]
^g^ Cu^2+^@Zr-MOF@TiO_2_ NRs	PEC	0.01–10	8.60	[35]
^h^ Pd/I:BiOBr-OVs/SOD-Au@PANI	PEC	0.0001–100	0.042	[36]
^i^ Ag_2_S/ZnO/ITO	PEC	0.00001–1	0.003	[37]
^j^ SiNWs@PDA	PEC	0.005–10	1.47	[38]
^k^ Ag@Cu_2_O core-shell SPs/TiO_2_/CdS	PEC	0.00002–50	0.0067	[39]

^a^ N-acetyl-L-cysteine-capped CdAgTe quantum dots and dodecahedral Au nanoparticles; ^b^ Manganese doped CdS sensitized graphene/Cu_2_MoS_4_ composite;**^c^** Carboxymethylated dextran-coated and gold-modified TiO_2_ nanotube arrays; ^d^ Nitrogen-doped carbon quantum dots–bismuth oxyiodide–flower-like SnO_2_; ^e^ ZnIn_2_S_4_/Bi_2_Se_3_ Nanocomposite; ^f^ N,S-GQDs and CdS co-sensitized hierarchical Zn_2_SnO_4_ cube; ^g^ Zr-MOF coated onto TiO_2_ NRs on fluorine-doped tin oxide; ^h^ Pd nanoparticles loading on the I-doped bismuth oxybromide with oxygen vacancies sensibilized by superoxide dismutase loaded on gold@polyaniline; ^i^ Ag_2_S/ZnO Nanocomposites. **^j^** Silicon nanowire arrays (SiNWs) at Polydiacetylene (PDA); ^k^ Ag at Cu_2_O core-shell submicron-particles on CdS QDs sensitized TiO_2_ nanosheets.

**Table 2:** Recovery values obtained from detection of cTnI in artificial blood plasma samples using the proposed immunosensor.

Sample	Additioned/ng mL^−1^	Found/ng mL^−1^	Recovery/%	RSD/%
**1**	0.05	0.049 (±0.001)	98.0	2.04
**2**	2.0	1.96 (±0.01)	98.0	0.51
**3**	50	49.24 (± 0.05)	98.5	0.10

## Data Availability

Not applicable.

## Supplementary Materials

The following supporting information can be downloaded at: https://www.mdpi.com/article/10.3390/bios13030379/s1, Figure S1. (A) Photoelectrochemical response of the Bi_2_S_3_/BiVO_4_/FTO platform obtained at different potentials. Amperometric measurements performed in 0.1 mol L^−1^ phosphate buffer (pH 7.4) containing 0.03 mol L^−1^ AA. (B) Plot of photocurrent vs. E_appl_. Data obtained from the Figure S1A; Figure S2. (A) Photoelectrochemical response of the Bi_2_S_3_/BiVO_4_/FTO platform obtained at different buffer solutions. (B) Plot of photocurrent vs. different buffer solutions. Amperometric measurements performed in 0.1 mol L^−1^ of buffer (pH 7.4) containing 0.03 mol L^−1^ AA. E_appl_ = 0 V vs. Ag/AgCl/KCl_sat_; Figure S3. (A) Photoelectrochemical response of the Bi_2_S_3_/BiVO_4_/FTO platform obtained at different AA concentrations (0.01–0.06 mol L^−1^). (B) Amperometric measurements performed in 0.1 mol L^−1^ phosphate buffer (pH 7.4) containing 0.04 mol L^−1^ AA. E_appl_ = 0 V vs. Ag/AgCl/KCl_sat_; Figure S4. Photoelectrochemical responses of the anti-cTnI/Bi_2_S_3_/BiVO_4_/FTO PEC immunosensor before (black amperogram) and after incubation with cTnI antigens (red amperograms) at different incubation times. The measurements were performed in 0.1 mol L^−1^ phosphate buffer, pH 7.4, containing 0.04 mol L^−1^ AA. E_appl_ = 0 V vs. Ag/AgCl/KCl_sat_. [anti-cTnI] = 5 µg mL^−1^; [cTnI] = 1 ng mL^−1^; Figure S5. Photoelectrochemical responses obtained with 5 (five) different anti-cTnI/Bi_2_S_3_/BiVO_4_/FTO PEC immunosensors under optimized conditions before (black amperograms) and after (red amperograms) incubation with cTnI. [cTnI]=1 ng mL^−1^, t_incubation_= 25 min; Figure S6. Photoelectrochemical responses obtained with the anti-cTnI/Bi_2_S_3_/BiVO_4_/FTO PEC immunosensor under optimized conditions before (black amperogram) and after (red amperogram) incubation with cTnI (1 ng mL^−1^) in absence and presence of different species (albumin, C-reactive protein, glucose, and myoglobin). [Foreign specie] = 100 ng ng mL^−1^; t_incubation_= 25 min.
