# Supplementary material for: Photoelectrochemical Determination of Cardiac Troponin I as a Biomarker of Myocardial Infarction Using a Bi2S3 Film Electrodeposited on a BiVO4-Coated Fluorine-Doped Tin Oxide Electrode
Source: Biosensors (Basel). 2023 Mar 13;13(3):379. doi: 10.3390/bios13030379 (PMC10046628; doi:10.3390/bios13030379)
Supplement: Supplementary file 1 [file biosensors-13-00379-s001.zip › biosensors-2244917-supplementary.pdf]

biosensors

MDPI

Supporting Information

Photoelectrochemical Determination of Cardiac Troponin I as a Biomarker of Myocardial Infarction Using a Bi_2S_3 Film Electrodeposited on a $BiVO_4$-Coated, Fluorine-Doped Tin Oxide Electrode

Thatyara Oliveira Monteiro [1], Antônio Gomes dos Santos Neto [2], Alan Silva de Menezes [3], Flávio Santos Damos [2], Rita de Cássia Silva Luz [2,*] and Orlando Fatibello-Filho [1,*]

[1] Department of Chemistry, Federal University of São Carlos, 13565-905 São Carlos, SP, Brazil
[2] Department of Chemistry, Federal University of Maranhão, 65080-805 São Luís, MA, Brazil
[3] Department of Physics, Federal University of Maranhão, 65080-805, São Luís, MA, Brazil
* Correspondence: rita.luz@ufma.br (R.C.S.L.); bello@ufscar.br (O.F.F.)

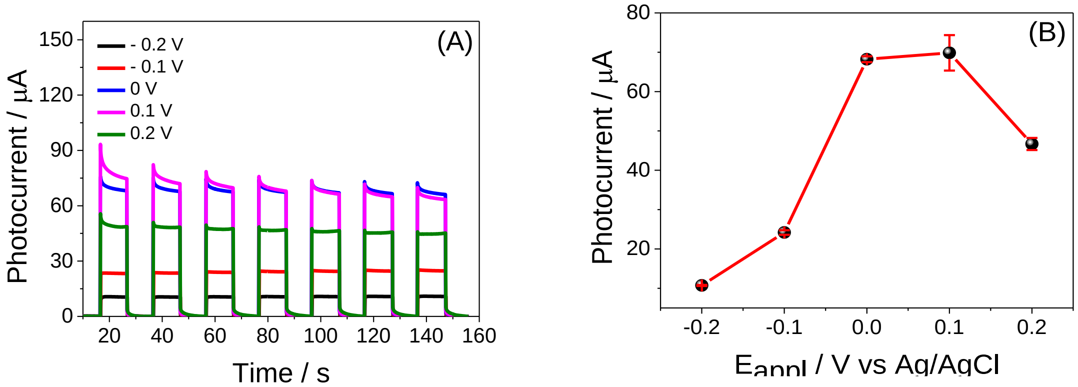

Figure S1. (**A**) Photoelectrochemical response of the $Bi_2S_3/BiVO_4$/FTO platform obtained at different potentials. Amperometric measurements performed in 0.1 mol L^{-1} phosphate buffer (pH 7.4) containing 0.03 mol L^{-1} AA. (**B**) Plot of photocurrent vs E_{appl}. Data obtained from the Figure S1A.

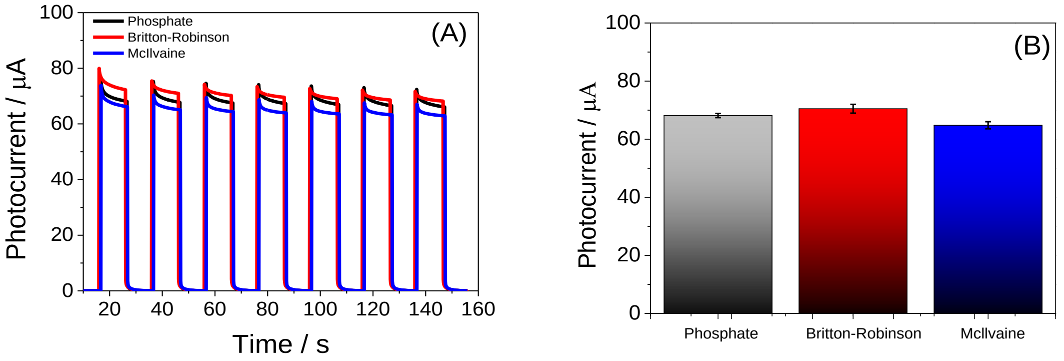

Figure S2. (**A**) Photoelectrochemical response of the $Bi_2S_3/BiVO_4$/FTO platform obtained at different buffer solutions. (**B**) Plot of photocurrent *vs*. different buffer solutions. Amperometric measurements performed in 0.1 mol L^{-1} of buffer (pH 7.4) containing 0.03 mol L^{-1} AA. E_{appl} = 0 V vs. Ag/AgCl/KCl_{sat}.

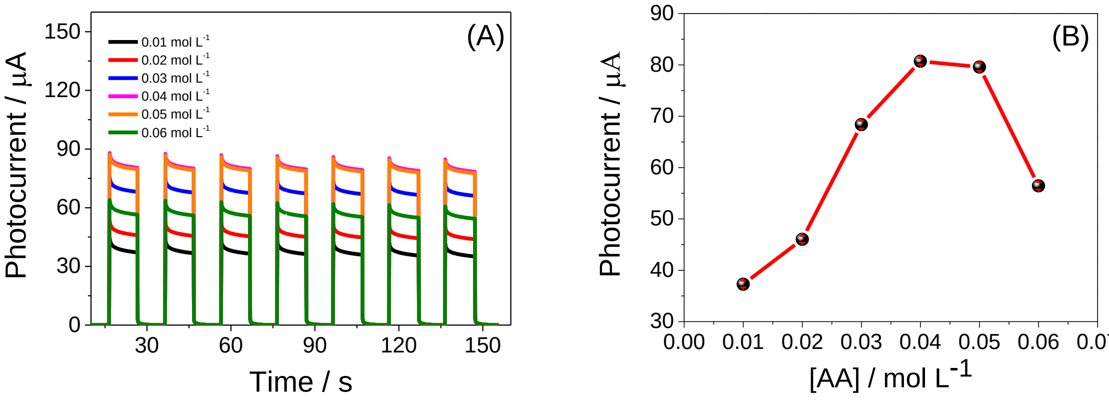

Figure S3. (**A**) Photoelectrochemical response of the $Bi_2S_3/BiVO_4$/FTO platform obtained at different AA concentrations (0.01–0.06 mol L^{-1}). (**B**) Amperometric measurements performed in 0.1 mol L^{-1} phosphate buffer (pH 7.4) containing 0.04 mol L^{-1} AA. E_{appl} = 0 V *vs*. Ag/AgCl/KCl_{sat}.

Data of Figure 3 C

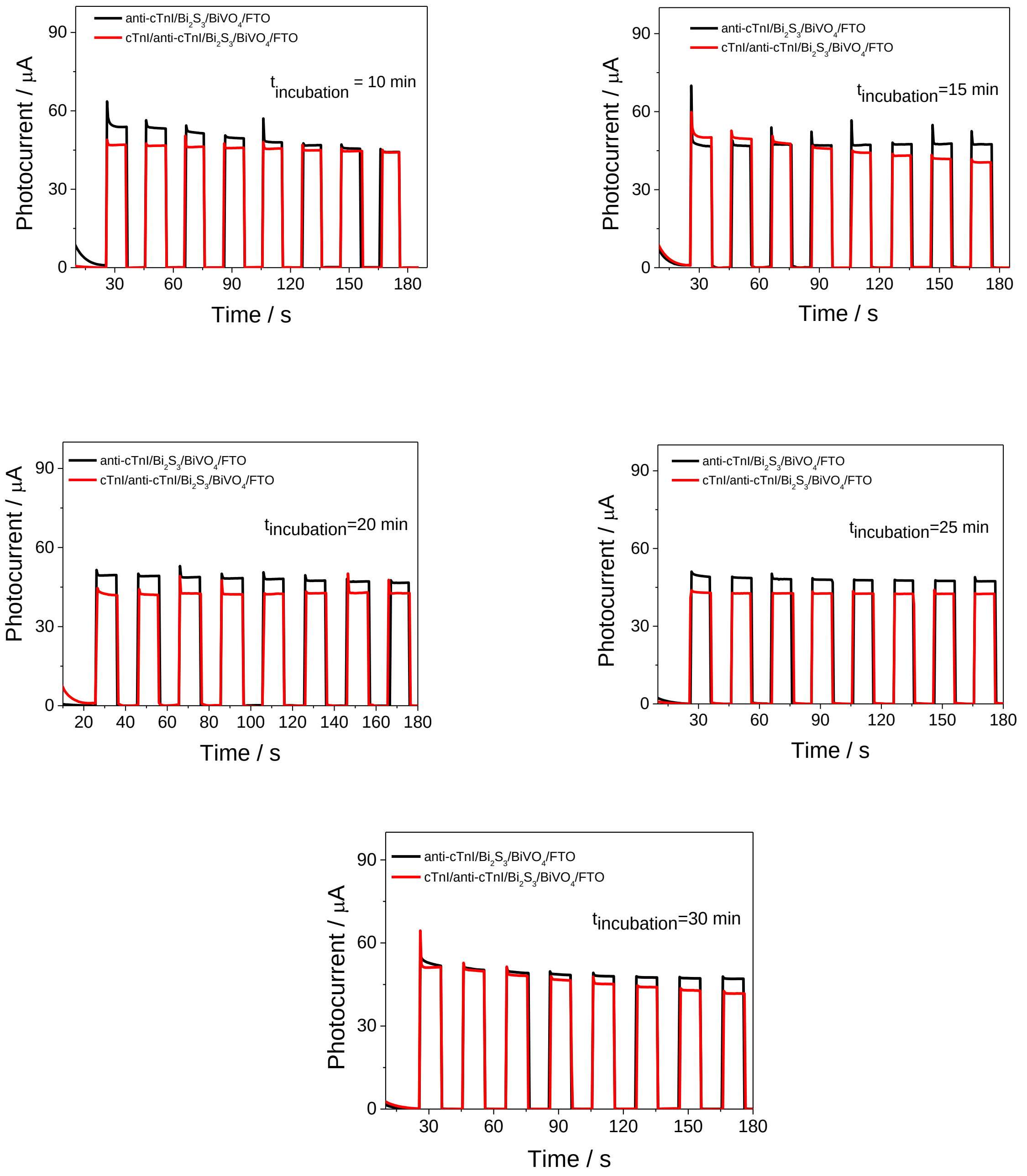

Figure S4. Photoelectrochemical responses of the anti-cTnI/Bi_2S_3/$BiVO_4$/FTO PEC immunosensor before (black amperogram) and afer incubation with cTnI antigens (red amperograms) at different incubation times. The measurements were performed in 0.1 mol L^{-1} phosphate buffer, pH 7.4, containing 0.04 mol L^{-1} AA. E_{appl} = 0 V *vs*. Ag/AgCl/KCl_{sat}. [anti-cTnI] = 5 µg mL^{-1}; [cTnI] = 1 ng mL^{-}.

Reproducibility (Data of Figure 5B)

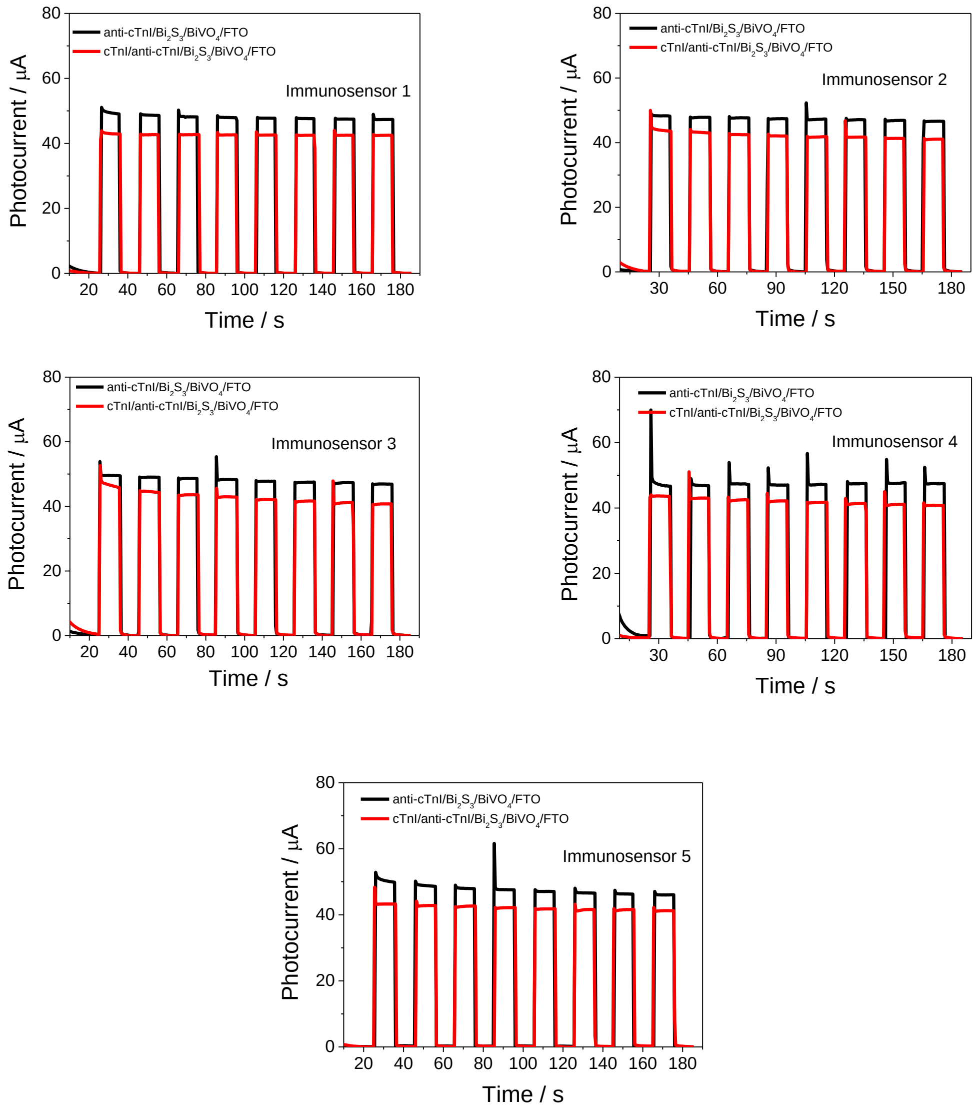

Figure S5. Photoelectrochemical responses obtained with 5 (five) different anti-cTnI/Bi_2S_3/$BiVO_4$/FTO PEC immunosensors under optimized conditions before (black amperograms) and after (red amperograms) incubation with cTnI. [cTnI]=1 ng mL^{-1}, $t_{incubation}$= 25 min.

Data of Figure 5C

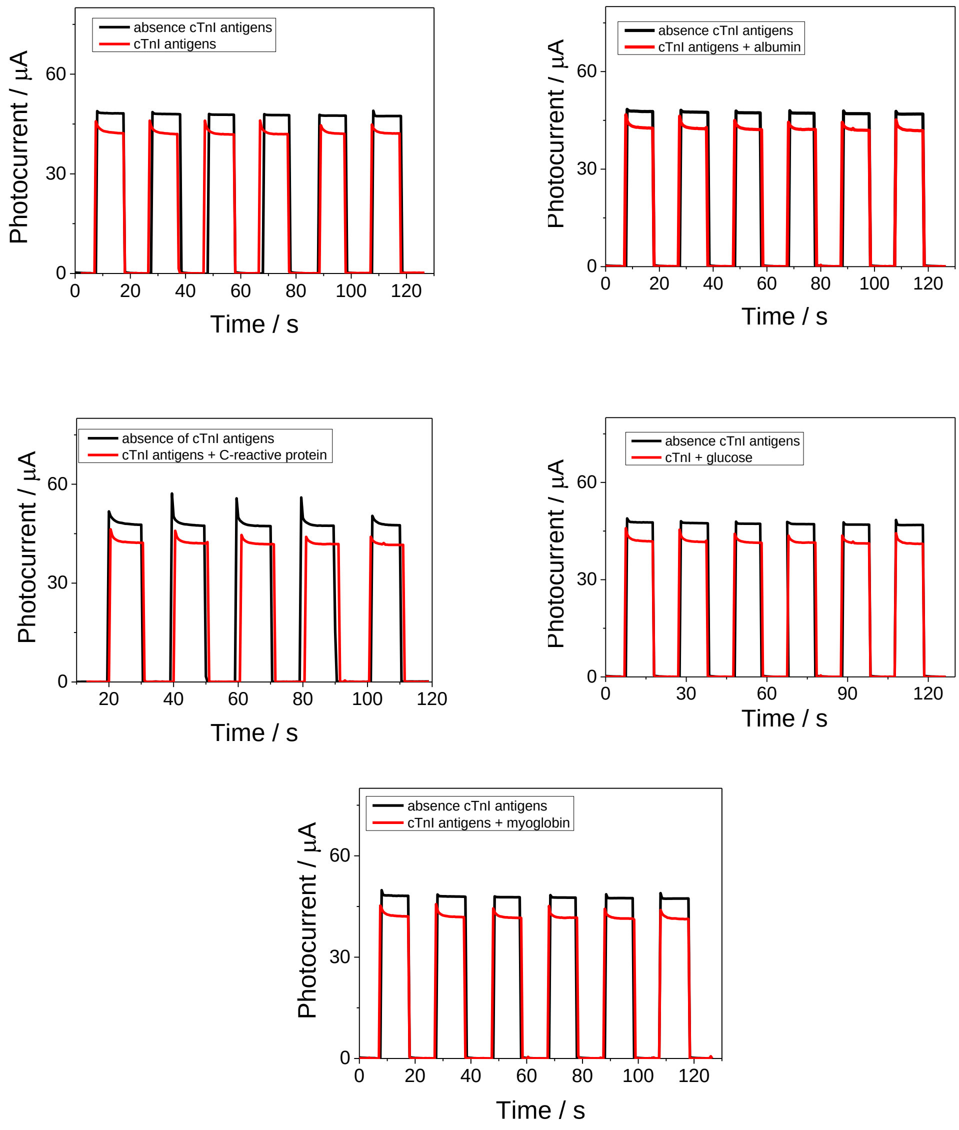

Figure S6. Photoelectrochemical responses obtained with the anti-cTnI/Bi_2S_3/$BiVO_4$/FTO PEC immunosensor under optimized conditions before (black amperogram) and after (red amperogram) incubation with cTnI (1 ng mL^{-1}) in absence and presence of different species (albumin, C-reactive protein, glucose, and myoglobin). [Foreign specie] = 100 ng ng mL^{-1}; $t_{incubation}$= 25 min.
